# Integrated treatment guided by RNA-seq–based endometrial receptivity assessment for infertility complicated by MEN1

**DOI:** 10.3389/fendo.2023.1224574

**Published:** 2023-10-20

**Authors:** Xi Huang, Jing Fu, Qiong Zhang, Jing Zhao, Zhongyuan Yao, Qiuping Xia, Hongying Tang, Aizhuang Xu, Aihua He, Shaolin Liang, Sijia Lu, Yanping Li

**Affiliations:** ^1^ Department of Reproductive Medicine, Xiangya Hospital, Central South University, Changsha, Hunan, China; ^2^ Clinical Research Center for Women’s Reproductive Health in Hunan Province, Changsha, Hunan, China; ^3^ Department of Reproductive Medicine Center, The Third Xiangya Hospital, Central South University, Changsha, Hunan, China; ^4^ National Comprehensive Utilization of Science and Technology Information Resources and Public Service Center, Scientific and Technical Information (STI)-Zhilian Research Institute for Innovation and Digital Health, Beijing, China; ^5^ ”Mobile Health” Ministry of Education-China Mobile Joint Laboratory, Xiangya Hospital, Central South University, Changsha, China; ^6^ Institute for Six-sector Economy, Fudan University, Shanghai, China; ^7^ Department of Clinical Research, Yikon Genomics Company, Ltd., Suzhou, Jiangsu, China

**Keywords:** endometrial receptivity, RNA-seq, PGT-M, personalized embryo transfer, MEN1

## Abstract

**Background:**

Preimplantation genetic testing (PGT) serves as a tool to avoid genetic disorders in patients with known genetic conditions. However, once a selected embryo is transferred, implantation success is attained independent of embryo quality. Using PGT alone is unable to tackle implantation failure caused by endometrial receptivity (ER) abnormalities in these patients.

**Methods:**

We validated our newly developed RNA-seq–based ER test (rsERT) in a retrospective cohort study including 511 PGT cycles and reported experience in treating an infertile female patient complicated by multiple endocrine neoplasia type 1 (MEN1).

**Results:**

Significant improvement in the clinical pregnancy rate was found in the performed personalized embryo transfer (pET) group (CR, 69.7%; *P* = 0.035). In the rare MEN1 case, pET was done according to the prediction of the optimal time of window of implantation after unaffected blastocysts were obtained by PGT-M, which ultimately led to a healthy live birth. However, none of the mRNA variants identified in the patient showed a strong association with the MEN1 gene.

**Conclusions:**

Applying the new rsERT along with PGT improved ART outcomes and brought awareness of the importance of the ER examination in MEN1 infertile female patients. MEN1-induced endocrine disorder rather than MEN1 mutation contributes to the ER abnormality.

**Trial Registration:**

Reproductive Medicine Ethics Committee of Xiangya Hospital Registry No.: 2022010.

## Introduction

Preimplantation genetic testing (PGT) is an important technique developed to select embryos during artificial reproduction technology (ART) and avoid embryonic genetic abnormalities that lead to miscarriage or the inheritance of genetic diseases. However, once a single unaffected embryo is transferred, high levels of implantation and live birth success are attained independent of patient age and embryo quality. Patients with known genetic conditions may still fail to establish a successful pregnancy due to damaged endometrial receptivity (ER) although they obtained normal embryos by PGT (1). As a relatively expensive process in China, ART treatment following multiple PGT attempts may place a significant financial burden on patient couples (2).

Better technologies for personalized embryo transfer (pET), which not only consider applying PGT to select the “right” embryo but also try to find the “right” time for implantation, may contribute to the improvement of implantation and live birth outcomes for patients with genetic disorders. To define the “right” time, there is a certain period of endometrial maturation, called ER (3), during which the trophectoderm of the blastocyst can attach to the endometrial epithelial cells and subsequently proceed to invade the endometrial stroma. In 2009, ER array (ERA) was developed to detect the specific time point in the endometrial cycle in which ER is optimal and embryo implantation is possible, so-called window of implantation (WOI) (4). The reliability and reproducibility of the ERA test for determining the exact time of the WOI, which can be used with better results than histological dating of ER, showed that it was accurate and consistent (5). However, a decade has passed since the launch of ERA, and there remains limited evidence of the optimal indication of ERA as conflicting effects are reported on obstetric outcomes (6, 7). To develop a more Asian-specific ER assessment tool with better clinical performance, our group applied an improved endometrial biopsy sampling scheme with a machine learning algorithm to construct a novel RNA-seq–based ER test (rsERT) consisting of ER-specific marker genes. The new method was initially validated in a cohort with 142 patients diagnosed with repeat implantation failure (8). This gives us the basic technology that we need to study the use of rsERT on a larger group of patients and to focus on some patients with rare genetic diseases like multiple endocrine neoplasia type 1 (MEN1).

MEN1 is a rare autosomal dominant condition (prevalence 3–20/100,000) resulting from mutations in the tumor suppressor gene *MEN1* and characterized by various neuroendocrine tumors such as parathyroid hyperplasia, pancreatic endocrine tumors, and pituitary adenomas. Patients with MEN1 may have amenorrhea and reproductive disorders due to hormonal abnormalities, but, currently, there are limited studies discussing the direct impact of *MEN1* mutation on fertility. There are only a few case reports that describe patients with MEN1 developing infertility as a further symptom of the disease (9–11), whereas a multigenerational cohort study of the MEN1 population (Tasman 1 MEN1 kindred) controversially indicated no adverse impact of MEN1 on patient fertility overall, but MEN1 may impair the reproductive potential of individuals with pituitary disease (12). Because it is unclear how MEN1 affects fertility, clinical guidelines for the disease, particularly therapy of patients with MEN1 with infertility, are restricted. In the current clinical setting, PGT-M is recommended to block birth defects such as monogenic gene diseases. However, the only reported successful PGT-M treatment for the MEN1 condition is for male patients (13). In terms of female patients, embryo transplantation may still fail because of defected ER and displacement of the time of ER considering the level of endocrine complication in patients with MEN1. In addition, even if the embryo is implanted, multiple endocrine disorders induced by MEN1 can have a long-term impact on maternal–fetal safety. Several attempts have been made to use multidisciplinary team (MDT) management for pregnant women who have a MEN1 diagnosis (14–16). Therefore, a combination of pET to ensure “successful” transplantation of *MEN1* mutation–negative embryo and MDT management to maintain homeostatic balance during the whole ART treatment may contribute to a positive treatment scheme for patients with MEN1.

To this end, we first further evaluated our newly developed rsERT in a retrospective cohort study including 511 PGT frozen embryo transfer (FET) cycles to see whether improved patient outcomes can be obtained by the combination of PGT with the rsERT application. Second, we applied the integrated treatment scheme to an infertile female patient complicated by MEN1 who had a failed pregnancy after PGT-M embryo transfer. A wide range of hormone levels in this patient were monitored and analyzed before and after we applied the treatment. Bioinformatic studies were performed to see whether any mRNA variants detected in this patient via rsERT are associated with the MEN1 mutation.

## Methods

### Subjects

In this retrospective cohort study, we evaluated the PGT FET cycle baseline characteristics and results in our center between April 2019 and May 2022. The main end-points were as follows: no pregnancy; blood β-human chorionic gonadotropin (β-HCG) below 10, 12 days after embryo transferred; and clinical pregnancy, accessed by ultrasound 28 days after transferred in β-HCG positive patients. In total, 511 FET cycles were included into the study and further divided into three groups: control group: only performed PGT, n = 432 cycles; pET group: the rsERT showed WOI displaced and pET performed, n = 33 cycles; and non-displaced group: rsERT showed normal WOI, n = 46 cycles. Among the three groups, the number of retrieval cycles in each group was 347, 31, and 35, respectively. Time to pregnancy (TTP) was defined as the duration (months) from patients obtained unaffected embryos to clinical pregnancy. In our retrospective study, we compared the cycles with clinical pregnancy rate (CPR), live birth rate (LBR), cumulative CPR (CCPR), and cumulative LBR (CLBR).

This study was conducted at the Department of Reproductive Medicine, Xiangya Hospital, Central South University. The study was approved by the Reproductive Medicine Ethics Committee of Xiangya Hospital (registration no. 2022010).

### Endometrium sampling

**Figure 1:**
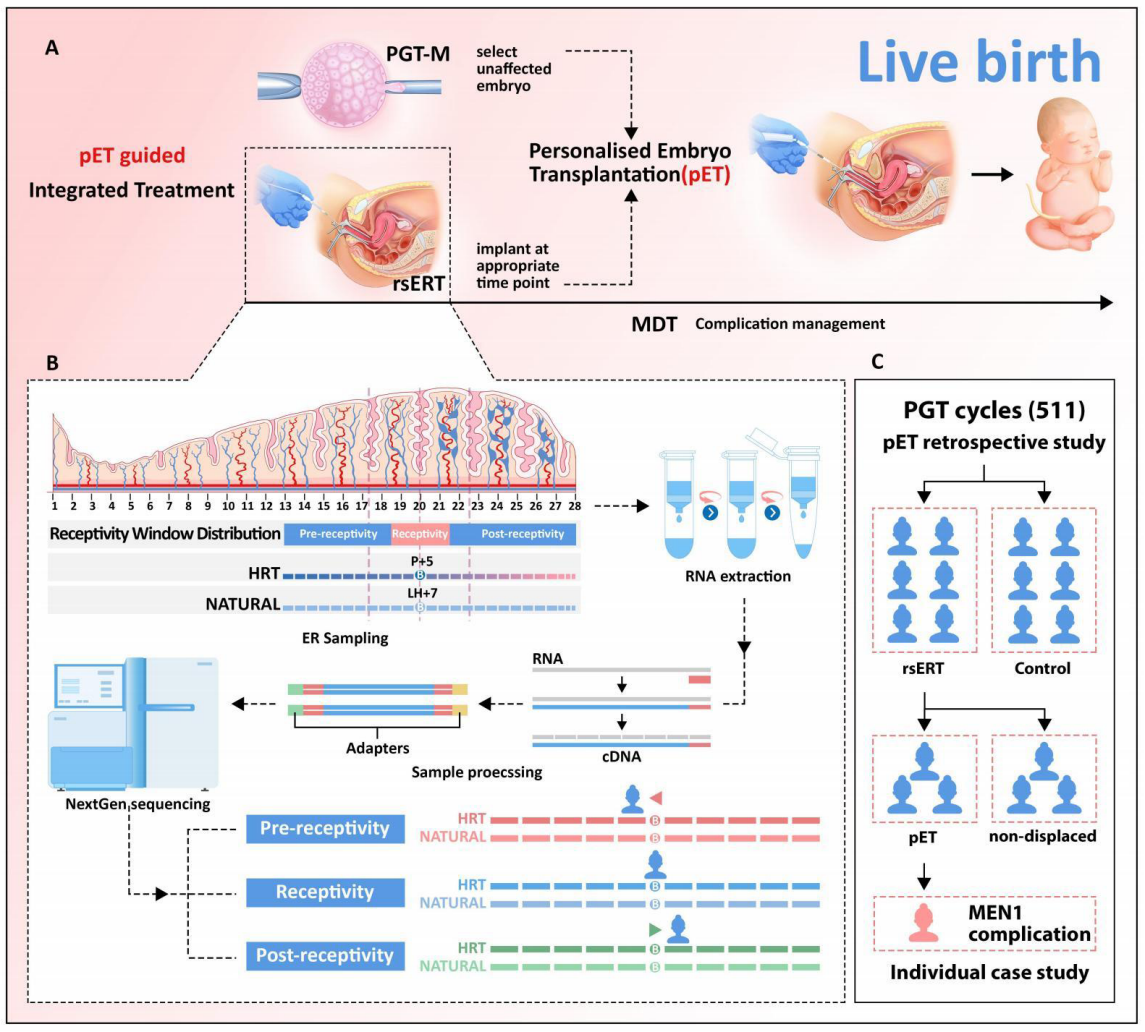
Validation of rsERT-guided integrated treatment scheme. **(A)** rsERT-guided integrated treatment scheme. The integrated treatment scheme is based on the combination of pET (PGT with rsERT) and MDT. **(B)** pET will be performed at the timing of optimal WOI predicted by rsERT, MDT will also be performed to ensure complication management if needed. **(C)** Data collected from 511 cycles treated in our center were divided into two major groups: performed PGT alone (control group) and rsERT group (further divided into pET and non-displaced groups).

The patient provided written informed consent that she understood that the endometrium biopsies were performed for research purposes. We sampled the endometrium on the sixth day of progesterone supplementation during the hormone replacement therapy cycle (defined as P + 5 day, where the first day as P + 0 day) (**Figure 1B**). The collected samples were immediately placed in 1.5 mL of RNAlater buffer, sealed, and cryopreserved at −80°C. Sequencing analysis was performed within 7 days of sampling.

### rsERT-guided personalized embryo transfer protocol

We described our development and how we guided pET according to the test result of rsERT in a previously published paper (8); pET was performed at the timing of optimal WOI predicted by rsERT. In brief, the endometrium RNA sequencing was put into the rsERT, and the method would predict the ER status of the sampling time. The timing of optimal WOI could be calculated according to the time of sampling time. The sample collected on P + 5 from this patient was predicted by rsERT model as pre-receptivity, and the optimal period of ER for her is 20 h after sampling. Subsequently, the corresponding frozen-thawed blastocysts transfer would be performed on the basis of this predicted time.

### Bioinformatics analysis

Z-values were calculated to analyze the differentially expressed genes (DEGs) between the patient’s endometrial mRNA and the background genes in the receptive phase of the rsERT. A *MEN1*-related gene list was obtained from STRING database. ToppGene Functional Annotation tool (ToppFun) was used to analyze the priority of DEGs based on the list. STRING was also used to analyze the protein–protein interaction (PPI) network among variants with *MEN1*. The sequence data reported in this study were archived in the Sequence Read Archive (SRA) with the accession number SUB13725948.

### Endocrine function monitoring

A systematic evaluation and adjustment of multiple endocrine functions by MDT management were performed, including patient’s pituitary function, thyroid function, parathyroid function, blood glucose, and lipid profile, by reproductive center physicians and endocrinologists before entering the cycle. Hormone level datasets before the treatment were obtained from the Department of Gynecology and Obstetrics in our affiliated hospital.

### Statistics

Continuous data subject to a normal distribution are presented as means ± SD and were compared using ANOVA analysis. Categorical data are expressed as counts and percentages and were compared using the Chi-square test or the Fisher’s exact test. A two-sided P-value equal to or less than 0.05 was considered to be statistically significant. Statistical analysis was performed using IBM SPSS software (version 25.0, IBM Corp.).

## Results

### Integrated treatment scheme and cycle characteristics

Here, we demonstrated a rsERT-guided integrated treatment scheme based on the combination of pET (PGT with rsERT) and MDT (**Figure 1A**). In this scheme, pET will be performed at the timing of optimal WOI predicted by rsERT, and MDT will also be performed to ensure complication management if needed. The predictive model of rsERT was developed and trained utilizing DEGs among the pre-receptive, receptive, and post-receptive endometrium collected from Asian female group (8) (**Figure 1B**). To validate the patient outcomes for the pET, we retrospectively analyzed the data collected from 511 PGT FET cycles treated in our center (**Table 1**). They were divided into two major groups: those who only performed PGT (control group) and those who also performed rsERT, with the rsERT group further subdivided into the pET group (rsERT result showed WOI displace, so PGT was also performed for pET) and the non-displace group (**Figure 1C**). There were no significant differences in the couple mean age, infertility type, infertility duration, PGT type, Anti Miillerian Hormone(AMH), endometrial thickness, endometrial pattern, and the percent of high-quality blastocysts. The number of oocytes retrieved in non-displaced group was more than pET group and control group (*P =* 0.026). The CPR in the pET group was significantly higher than that in the control group and non-displaced group (*P =* 0.035). The previous cycle numbers in non-displaced group were more than that in the pET group and the control group (*P* < 0.001), whereas there was no significant difference in TTP (*P =* 0.550) (**Table 1**). Multiple comparisons tests (pairwise group comparisons used a Bonferroni-adjusted significance level of.017) were also performed; the result showed the CPR in the pET group was significantly higher than that in the control group (*P* < 0.011 for pET vs. control) and that in the non-displaced group did not differ significantly with both control and pET (*P =* 0.484 for non-displaced vs. control, *P =* 0.118 for non-displaced vs. pET).

**Table 1:** Characteristics and clinical pregnancy rates of the rsERT and control groups.

Characteristics		rsERT		*P*-value
	Control	pET	Non-displaced	
	(n = 432)	(n = 33)	(n = 46)	
Female age (means ± SD), years	32.10 ± 4.56	31.91 ± 4.79	31.91 ± 4.63	*P =* 0.943
Male age (means ± SD), years	35.7 ± 7.05	36.03 ± 7.52	34.65 ± 7.15	*P = 0.590*
Infertility type, N (%)				
Primary infertility	109 (25.2%)	14 (42.4%)	15 (32.6%)	*P =* 0.067
Secondary infertility	323 (74.8%)	19 (57.6%)	31 (67.4%)	
Infertility duration (means ± SD), years	3.14 ± 3.00	3.70 ± 3.45	3.04 ± 2.65	*P =* 0.567
PGT type, N (%)				
PGT-A	220 (50.9%)	13 (39.4%)	25 (54.3%)	*P =* 0.628
PGT-SR	143 (33.1%)	15 (45.5%)	15 (32.6%)	
PGT-M	69 (16.0%)	5 (15.2%)	6 (13.0%)	
AMH (means ± SD), ng/mL	3.91 ± 2.98	4.59 ± 3.08	3.40 ± 2.15	*P =* 0.219
Number of oocytes retrieved	14.4 ± 7.758	16.727 ± 6.8706	17.087 ± 7.4441	***P =* 0.026**
(means ± SD)				
				*P =* 0.094^a^
				*P =* 0.837^b^
				***P =* 0.024^c^**
Endometrial thickness,	9.394 ± 1.7819	9.403 ± 1.6124	9.791 ± 2.0764	*P =* 0.363
(means ± SD), mm				
Endometrial pattern, N (%)				
A	100 (23.1%)	12 (36.4%)	15 (32.6%)	*P =* 0.069
B	296 (68.5%)	21 (63.6%)	25 (54.3%)	
C	36 (8.3%)	0 (0.0%)	6 (14.3%)	
Proportion of high-quality blastocysts, N (%)	414 (95.8%)	33 (100.0%)	45 (97.8%)	*P =* 0.402
Previous cycle numbers, (means ± SD)	2.72 ± 1.101	3.48 ± 1.787	3.59 ± 1.833	***P =* 0.000**
				*P =* 0.062^a^
				*P =* 0.992^b^
				***P =* 0.009^c^**
CPR, N (%)	202 (46.8%)	23 (69.7%)	24 (52.2%)	***P =* 0.035**
				***P =* 0.011** ^a^
				*P =* 0.118^b^
				*P =* 0.484^c^
TTP, means ± SD, month	6.05 ± 6.54 (n = 202)	6.91 ± 3.91 (n = 23)	7.38 ± 6.45 (n = 24)	*P =* 0.550
LBR, N (%)	172 (39.9%)	16 (48.5%)	20 (43.5%)	*P =* 0.581

Bold P-value indicates statistical significance; CPR, clinical pregnancy rate; TTP, time to pregnancy; LBR, live birth rate; a, indicating the p-value of the pET group compared with the control group; b, indicating the p-value of the pET group compared with the non-displaced group; c, indicating the p-value of the non-displaced group compared with control group.

### Improvement of patient outcome

There was no significant statistical difference in the baseline clinical characteristics among the three groups. rsERT was performed in some patients to accurately determine whether the endometrium was in the WOI and, after pET, was performed in patients with abnormal ER, and the CPR was obviously improved. The LBR in the pET group was 8.6% higher than that in the control group, and the CCPR and CLPR were both higher in the pET group than that in the control group, although the difference was not statistically significant (**Table 2**). Furthermore, there was not a statistically significant difference in TTP among groups with CPR, which means that there was not a delay in getting patients ready for pregnancy; although the pET technique took slightly longer, it increased the probability that they would get pregnant.

**Table 2:** CCPR and CLBR of the rsERT and control groups.

		rsERT		*P*-value
Characteristics	Control	pET	Non-displaced	
	(n = 347)	(n = 31)	(n = 35)	
CCPR, N (%)	200 (57.6%)	23 (74.2%)	22 (62.9%)	P = 0.180
CLBR, N (%)	172 (49.6%)	16 (51.6%)	16 (54.3%)	P = 0.855

CCPR, cumulative clinical pregnancy rate; CLBR, cumulative liver birth rate.

### Rare MEN1 patient complication

**Figure 2:**
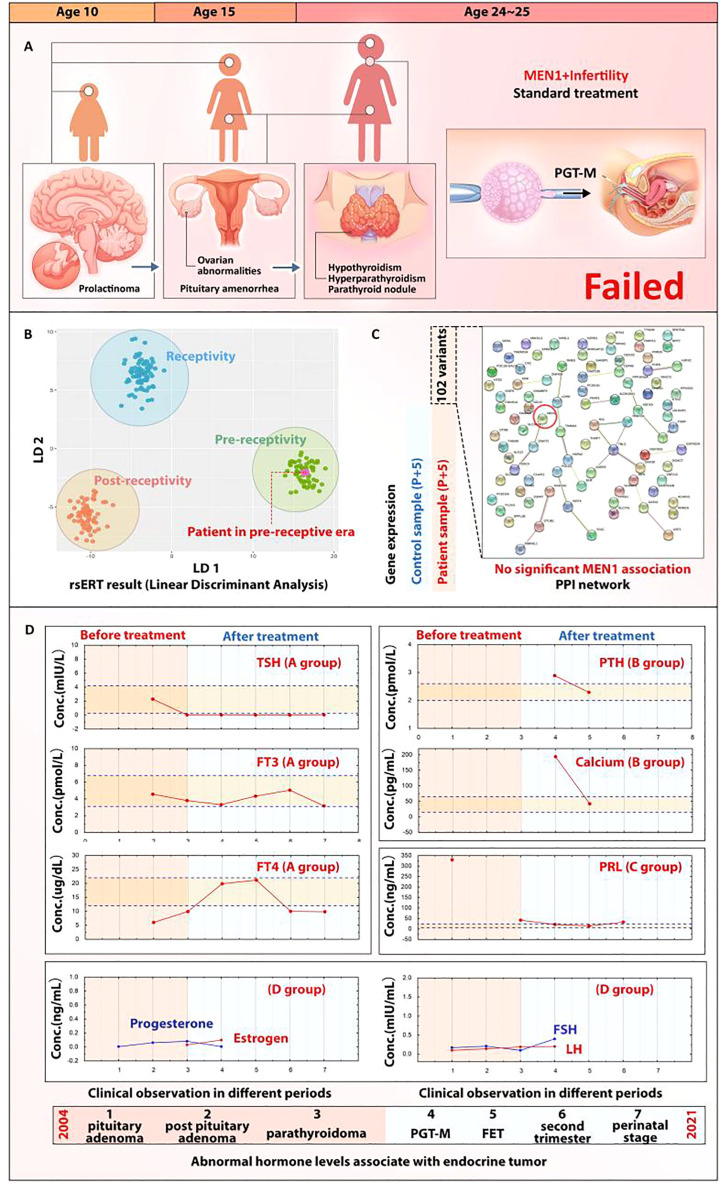
MEN1 and ER abnormality. ER abnormality in this reported case may be induced by the MEN1-associated endocrine conditions but not MEN1 mutation. **(A)** Indication of the rsERT biopsy day and presentation of rsERT result; the purple dot shows that the patient’s sample was in the pre-receptive area. **(B)** rsERT result for the patient with MEN1. **(C)** PPI analysis of MEN1 with the 102 variants identified; no strong association can be observed. **(D)** Restoration of endocrine homeostasis of the patient; hormones maintained a normal level under MDT management.

Within the 511 PGT FET cycles cohort, there was a rare infertility patient case complicated by MEN1. The patient was a 26-year-old Chinese woman. She discovered a large prolactinoma in her brain in 2004 (age 10), which later received radiotherapy and oral bromocriptine treatment after the resection was completed. She was diagnosed with pituitary amenorrhea in 2009 (age 15) and was given oral medication to establish an artificial menstrual cycle. In 2018 (age 25), she developed symptoms of central hypothyroidism, hyperparathyroidism (HPT), and a parathyroid nodule. Later, she was diagnosed with MEN1 after a mutation of the MEN1 gene c1268G>A (p.Trp423Term) was identified by whole exon sequencing, which also indicated the mutation came from her father. The patient then underwent parathyroidectomy twice and received postoperative hormone replacement therapy. Since her marriage in 2017 (age 24), she has not been able to conceive. Therefore, she decided to accept PGT-M treatment in 2019 (age 25) with her husband. The couple got three blastocysts after intracytoplasmic sperm injection (ICSI) and blastocyst culture. PGT-M revealed that only one of them did not carry pathogenic gene mutation, but biochemical pregnancy occurred after the normal blastocyst was implanted (**Figure 2A**). Endocrine abnormalities including hypopituitarism, obesity, central hypothyroidism, primary HPT, and hyperlipidemia were found at the time that the patient was admitted to our center in 2020 (age 26) for in vitro fertilization (IVF) treatment. As a first step in this case, we managed the patient’s weight loss and restored her hormone balance. A controlled ovarian hyperstimulation protocol without pituitary downregulation was used, and 11 oocytes were obtained. Four blastocysts formed after ICSI, and two of them were found to be euploid embryos without maternal pathogenic genes after PGT-M. The patient’s ER was examined using rsERT, and a pET for the unaffected embryo to the patient was performed on the basis of the rsERT result (**Figure 2B**). After pET, progesterone for luteal support was administered daily, including 600 mg intravaginal and 200 mg oral. Oral medications, such as Bromocript 2.5 mg quaque die (Qd) for hyperprolactinemia, Euthyrox (Merck) 112.5 µg (Qd) for hypothyroidism, metformin 0.5 g bis in die (Bid) for insulin resistance, were used long-term under MDT monitoring. Twenty-eight days later, the ultrasound result confirmed the intrauterine pregnancy. The patient had a cesarean section at 38 + 2 weeks’ Gestational age (GA) and delivered a live male infant with a weight of 3,850 g. Apgar scores were 10 for 1 min, 5 min, and 10 min after birth.

### Characterization of MEN1 mutation association

To further study whether the *MEN1* mutation directly impacts the ER at the genetic level, we identified 102 mRNA variants in the patient’s mRNA profile and found out that *GATA2* and *NR4A2* ranked the top two genes (**Additional file 1**). However, when we queried the STRING database for PPI analysis, no strong association of *MEN1* with the profile (weak association score of 0.621) was found for *ZNHIT2*, ranking the 41st in the gene cluster (**Figure 2C**). As no association could be found with the MEN1 mutation, we posited that the ER abnormality may be induced by the long-term impact on the endocrine disorders in this patient caused by MEN1, as pituitary prolactinoma and parathyroid adenoma were two of the major clinical phenotypes. The patient’s historical clinical data showed a serious hormonal imbalance since the age of 10. By applying MDT management after the admission to the center, most of the hormones’ level were restored to a normal range (**Figure 2D**) and the restoration contributed to the success rate of personalized embryo transplantation.

### Live birth achieved by integrated treatment

**Figure 3:**
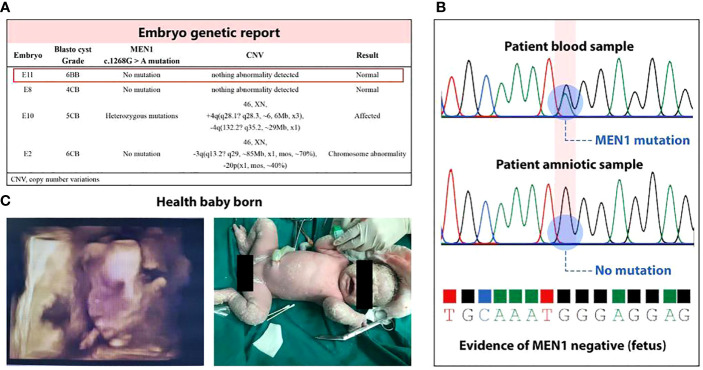
Health live birth. **(A)** Unaffected blastocysts were obtained by PGT-M; E11 was selected on the basis of its higher blastocyst grade in Gardner score system. **(B)** Amniocentesis showed no abnormalities in fetal chromosomes and no MEN1 c.1268G>A mutation was detected. **(C)** A baby was born with Apgar score 9-10-10, without positive airway pressure support for respiratory distress. The infant reached his developmental milestones by his age.

We performed pET with MEN1 mutation–negative blastocyst (**Figure 3B**) based on the estimated WOI. Thirty-five days after transplantation, the patient was sent to the Department of Obstetrics for further MDT management. No abnormalities of fetal nuchal translucency were observed at 12 weeks’ GA, and then oral metformin of the patient was discontinued. Amniocentesis, which was performed in the same month, showed no abnormalities in fetal chromosomes, and no MEN1 c.1268G>A mutation was detected (**Figure 3A**). The patient had a cesarean section at 38 + 2 weeks’ GA and delivered a live male infant with a weight of 3,850 g (**Figure 3C**).

## Discussion

Considering that the clinical outcomes of the application of ERA remain under debate, we aim at developing a more suitable ER assessment method for Asians, so we established the rsERT by utilizing Chinese patient samples collected in our center. In our study, PGT for aneuploidy (PGT-A) was the most component of PGT type. It analyzes the chromosome copy number to diagnose the aneuploids embryos. In ART, PGT-A is usually applied to patients with advanced maternal age, recurrent pregnancy loss, and recurrent implant failure. PGT-M helps select embryos free from monogenic disorders, reducing the risk of transferring embryos with genetic issues that could hinder implantation. The combination of rsERT and PGT creates more personalized and precise treatment plans for each individual patient. This approach considers both the genetic health of embryos and the receptivity of the endometrium, potentially leading to improved outcomes.

To further explore the patient benefit within the group of people who underwent PGT, our retrospective study result indicates performing rsERT along with PGT can largely increase patient benefit as transferable embryos are difficult to obtain (especially considering the MEN1 case): CPR was significantly higher compared to the control group; the LBR, CCPR, and CLPR were all higher in the pET group than that in the control group. Although statistical significance of CPR may not have been observed in the pET group and the non-displaced group in certain cases, LBR, CCPR, and CLPR showed no statistical difference; it is possible that the limited sample size could have resulted in insufficient statistical efficiency. We assumed that this still be an optimistic tendency. In the future study, we plan to enhance statistical efficiency by increasing the sample size, optimizing experimental design, and considering other factors that might influence statistical efficiency. This will enable us to detect potential differences and provide more convincing support for the research findings more accurately. Actually, we are already performing a multi-center randomized clinical trial to obtain more credible data.

Although performing rsERT slightly increases the TTP, it could be saved by increasing the success rate of implantation. The findings of the retrospective analysis give us the confidence to administer the integrated treatment to the patient with unique MEN1.

There are a limited number of clinical guidelines covering the treatment and management of patients with MEN1 with infertility. The current MEN1 clinical practice, which is generally similar to tumors occurring in non-MEN1 patients, suggests performing genetic tests for index patients with MEN1 and their first-degree relatives (17). Therefore, the application of PGT-M can be seen in a few MEN1-related IVF treatment cases to block the MEN1 mutation inheritance. However, the carriers of MEN1 syndrome in those reports were male patients. Whereas in our case, the 25-year-old female patient received an unaffected blastocyst via PGT-M but underwent an unsuccessful embryo transfer, which brings our awareness of exploring further treatment schemes in addition to PGT-M for patients with MEN1 with infertility.

This patient harbored a mutation of the *MEN1* gene c.1268G>A and presented with pituitary adenoma as well as parathyroid. A similar mutation variant has been reported in an Australian case, who presented with clinical phenotypes including lung and thymic carcinoids, prolactinoma, nonfunctioning pituitary adenomas, insulinomas, and HPT (18). The literature search indicated that *MEN1* may affect ER via decidualization by interfering with estrogen alpha receptor *ESR1* (19) and nuclear factor–κappa B (NF-κB) (20). This finding encouraged us to investigate the patient’s ER condition, and, thus, we applied rsERT, an RNA-seq–based ER tool that is capable of identifying ER-related genes and predicting the optimal WOI for pET.

The rsERT result showed that the WOI of the patient was delayed, but, interestingly, both *ESR1* and NF-κB could not be found in the patient’s mRNA variants, whereas two other genes, *NR4A* and *GATA2*, were found to rank the top two in the gene cluster. Our expectation might have been that MEN1 may have had a direct impact on ER. In such a case, either *ESR1* and NF-κB can be found in the mRNA variants or the association of *MEN1* with *NR4A* and *GATA2* can be identified. However, the PPI analysis showed a negative result in all the cases mentioned. Given the achievement of clinical pregnancy via pET, which is dependent on ER prediction and WOI identification, our finding adds to the scientific debate on whether MEN1 affects infertility by providing limited evidence that MEN1 has no direct effect on ER but is impaired ER by endocrine disorders. In addition, this finding also emphasizes the importance of performing MDT management for patients with MEN1 with infertility to maintain endocrine homeostasis. It also provides us the new research idea and the clinical application of rsERT in the future.

## Conclusions

The combination of rsERT and PGT contributes to a pET that assists clinicians in selecting the “right” blastocyst and performing embryo transfer at the “right” time, and the MDT management plays an important role in maintaining endocrine homeostasis. The case elucidates a new angle for developing treatment guidelines for infertile patients with MEN1 by applying an integration of multiple techniques, including rsERT, PGT-M, and MDT management.

## Data availability statement

The original contributions presented in the study are included in the article/supplementary material. Further inquiries can be directed to the corresponding authors.

## Ethics statement

The studies involving human participants were reviewed and approved by the Reproductive Medicine Ethics Committee of Xiangya Hospital (Registry No.2022010). The patients/participants provided their written informed consent to participate in this study. Written informed consent was obtained from the individual(s) and minor(s)’ legal guardian/next of kin for the publication of any potentially identifiable images or data included in this article.

## Author contributions

YL, XH, and JF formulated the study and collected the data. SSL, XH, and JF analyzed the data, edited the manuscript, and organized the figures and tables. YL, QZ, JZ and AX were involved in the clinical treatment team. SJL and AH performed the endometrium sampling and RNA sequencing. ZY and QX performed the embryos PGT. HT was involved in following-up the patient. XH and JF are co-first authors; the two authors contributed to this article equally. All authors contributed to the article and approved the submitted version.
